# Decoding the Effect of Frailty vs. Physiologic Age in Octogenarian and Nonagenarian Colectomy Outcomes for Colon Cancer

**DOI:** 10.3390/jcm14175985

**Published:** 2025-08-24

**Authors:** Philip Drohat, Alexandra E. Hernandez, Ana M. Reyes, Karishma Kodia, Chelsea Caplan, Talia R. Arcieri, Shayan Khalafi, Matthew S. Meece, Vanessa W. Hui

**Affiliations:** 1 Miller School of Medicine, University of Miami, Miami, FL 33136, USA; 2 Dewitt Daughtry Family Department of Surgery, University of Miami Miller School of Medicine, Miami, FL 33136, USAanamreyes2022@gmail.com (A.M.R.);; 3 Department of Colorectal Surgery, Cleveland Clinic Florida, Weston, FL 33331, USA; 4 Allegheny Health Network, Pittsburgh, PA 15212, USA

**Keywords:** frailty, octogenarian, nonagenarian, elderly patients, colorectal cancer, colectomy, post-operative complications, surgical outcomes, risk stratification

## Abstract

**Background/Objectives**: Colorectal surgeons continue to care for an aging cancer population with increasing comorbidities and frailty. Frailty, characterized by a systemic physiologic decline associated with aging, is an increasingly popular focus in surgical outcomes research. This retrospective study investigates how frailty impacts outcomes in the octogenarian and nonagenarian populations undergoing surgical treatment for colon cancer. **Methods**: Data from the National Surgical Quality Improvement Program (NSQIP) colectomy-targeted variables dataset from 2015 to 2021 were utilized for this analysis, including patients 80 years of age and older. Frailty was assessed using the five-factor modified frailty index (mFI-5). The study examined post-operative outcomes across frailty groups in this population. **Results**: From 2015–2021, there were 10,671 patients aged 80 years and older who underwent colectomy for colon cancer, of whom 1259 (11.8%) were 90 years or older and 2844 (26.7%) were severely frail. Frailty significantly impacted post-operative colectomy outcomes in this population. On univariate analysis, frail patients had higher rates of pneumonia (*p* = 0.015), unplanned intubation (*p* = 0.012), stroke (*p* < 0.001), myocardial infarction (*p* = 0.011), readmission (*p* < 0.001), long length of stay (*p* < 0.001), and mortality (*p* < 0.001) compared to non-frail patients. On multivariate analysis, severe frailty (mFI-5 of 2 or more) was associated with an increased odds of unplanned intubation (aOR 2.41, 95% CI 1.27–4.59), long length of stay (aOR 1.73, 95% CI 1.44–2.09), readmission (aOR 1.84, 95% CI 1.42–2.39), and mortality (aOR 1.95, 95% CI 1.20–3.15) compared to non-frail patients. **Conclusions**: Frailty plays a critical role in influencing the outcomes of octogenarians and nonagenarians undergoing colectomy for colon cancer within the NSQIP dataset. Future work should investigate whether addressing frailty prior to surgery in this population can improve patients’ post-operative courses.

## 1. Introduction

Medical advancements have increased life expectancy, causing surgeons to more frequently encounter elderly populations with complex health challenges. Elderly individuals are more likely to have complex comorbidities and frailty, defined as the systemic physiologic decline associated with aging [1,2]. Frailty is highlighted in the surgical literature as an important determinant of post-operative outcomes, and is a better predictor of morbidity and mortality than chronological age [3]. While age carries increased risk for post-operative complications and mortality, people of the same chronological age can have vastly different risk profiles. Alternatively, frailty is an estimate of one’s physiologic reserve and resilience to stressors, making it a more favorable predictor of a patient’s risk in the post-operative period [3,4]. Generally, frail patients are at risk of post-operative complications and worse outcomes; however, less is known about how frailty impacts the very elderly population. Understanding these impacts of frailty in the very elderly may allow surgeons to educate patients on the risks of surgery with greater accuracy and to identify targets for optimization in the treatment of very elderly patients undergoing surgery for colon cancer.

Given the rapidly aging population, discussion of surgical care of the elderly and very elderly is becoming increasingly important, and surgical care of the elderly patient with colon cancer should be no exception. As of 2017, individuals over 80 years old accounted for 18% of male and 27% of female colorectal cancer diagnoses, but accounted for 27% and 40% of all colorectal cancer deaths, respectively [5]. Despite this overrepresentation in mortality, focused discussion of octogenarians and nonagenarians is underrepresented in the colon cancer literature, indicating a need to identify specific risk factors for morbidity and mortality in this population. Additionally, there is a need to understand the impacts of frailty in this very elderly population, given that frail patients with colorectal cancer treated with curative intent have worse oncologic and survival outcomes after controlling for age [6].

Studies have demonstrated that frail patients have increased rates of post-operative hemorrhage and infection, longer lengths of stay, discharge to assisted living facilities, and mortality [7,8]. Frail patients undergoing colectomy for colon cancer experience increased rates of infections, cardiopulmonary complications, anastomotic leakage, and numerous other complications, as well as increased mortality; however, the impact of frailty on specific post-operative complications in octogenarians and nonagenarians has not been studied [9]. This lack of age-specific, frailty-informed data limits surgeon’s ability to accurately counsel their patients and family members about surgical candidacy. A better understanding of how frailty affects post-colectomy outcomes in the very elderly would provide physicians with more appropriate tools to effectively risk stratify their patients. This could allow for increased shared decision making, more comprehensive and tailored treatment plans, and improved outcomes in this susceptible population [10]. Understanding specific complications more likely to occur in the frail, very elderly population is critical for gaining insight into how to best medically optimize patients pre-operatively and take preventative measures in their post-operative care.

The objective of this study was to investigate the impact of frailty on specific post-operative complications after colectomy for colon cancer in the octogenarian and nonagenarian population. The broad intention of this work is to provide insight into patient selection and areas of potential pre- and post-operative optimization for these patients.

## 2. Materials and Methods

### 2.1. Population and Data

The 2015–2021 American College of Surgeons National Surgical Quality Improvement Program (NSQIP)-targeted colectomy database was used to identify octogenarian and nonagenarians undergoing any elective colectomy for colon cancer. NSQIP provides patient data from more than 400 hospitals, including demographics, outcomes, and pre-operative, intra-operative, and post-operative variables for major surgical procedures. The database benefits from several unique features of its data collection, including trained data collectors, standardized variable definitions, and the diverse range of participating hospitals.

We included all patients aged 80 years or older undergoing elective operation for colon cancer with or without obstruction. Of note in NSQIP, all patients who are 90 years or older are assigned an age of 90 years to avoid potential identification of patients. We excluded patients who underwent an emergent operation, those with known metastatic disease prior to surgery, and those with unknown metastatic status prior to surgery. We also excluded patients with hospital lengths of stay longer than 30 days given that NSQIP does not include complications occurring beyond 30 days of admission regardless of discharge status.

Variables collected included age, sex, self-reported race and ethnicity, body mass index (BMI), comorbidities, American Joint Committee on Cancer 8th Edition clinical stage (AJCC clinical stage), pre-operative mechanical bowel preparation, pre-operative oral antibiotic administration, chemotherapy within the last 90 days, operative approach, and total operative time.

Frailty was measured using the modified five-factor frailty index (mFI-5), which assigns one point to each of the following comorbidities: diabetes mellitus (DM), congestive heart failure (CHF), chronic obstructive pulmonary disease (COPD), hypertension requiring medication, and non-independent functional status [11]. Frailty was subsequently categorized for analysis as non-frail (mFI-5 of 0), mildly frail (mFI-5 of 1), and severely frail (mFI-5 of 2 or more). These groups are based on the prior literature [12,13]. Race was categorized into four groups (Asian American and Pacific Islander (AAPI), Black, Other, and White). “Other” included American Indian or Alaska Native and patients with multiple races, other race, or unknown race. BMI was categorized as follows: underweight (<18.5), normal weight (18.5–24.9), overweight (25.0–29.9), and obese (>29.9). AJCC clinical stage was calculated using the provided values for pathologic T, N, and M staging.

We analyzed univariate differences in rates of the following post-operative complications between frailty groups: mortality, readmission, unplanned re-operation, long length of stay (defined as a hospital stay longer than 8 days), anastomotic leak, prolonged nasogastric tube, superficial incisional surgical site infection (SSI), deep incisional SSI, organ/space SSI, sepsis, septic shock, Clostridioides difficile (C. diff) infection, wound disruption, pneumonia, unplanned intubation, pulmonary embolism (PE), mechanical ventilation for longer than 48 h, acute renal failure, urinary tract infection (UTI), stroke or cerebrovascular accident (CVA), cardiac arrest requiring cardiopulmonary resuscitation (CPR), myocardial infarction (MI), intra-operative or post-operative blood transfusions, and deep vein thrombosis (DVT) requiring therapy. Cut-off for length of stay was chosen given that 8 days was the 3rd quartile of hospital length of stay (median 5 days, interquartile range 3 to 8 days).

We performed multivariate analysis to test for an independent association of frailty and age group with long length of stay, unplanned re-operation, unplanned intubation, readmission, and mortality while controlling for covariates. 

### 2.2. Statistical Analysis

Univariate analyses were performed using chi-square tests for association for categorical variables, and the Mann–Whitney U test for total operation time, which was a nonparametric continuous variable. Multivariate analyses were performed with multivariable logistic regression and included variables with a *p*-value of less than 0.2 in the chi-square or Mann–Whitney U tests. Adjusted odds ratios (aORs) and 95% confidence intervals (CIs) were calculated. Multiple comparisons were adjusted for using a Bonferroni correction for five outcomes, with adjusted statistical significance set at α < 0.01. All analyses were conducted using IBM SPSS Statistics (Version 28).

## 3. Results

### 3.1. Patient Factors by Frailty Group and Age Group

Of the 10,671 patients included in our analysis, 9412 (88.2%) were octogenarians and 1259 (11.8%) were nonagenarians. In terms of frailty, 2345 (22.0%) were non-frail, 5482 (51.4%) were mildly frail, and 2844 (26.7%) were severely frail. Table 1 presents all patient factors categorized by frailty group. Sex distribution varied significantly across groups, with the proportion of males greatest in the severely frail group (*p* < 0.001). Regarding race, most patients in all groups were White, with proportions of White patients decreasing as frailty increased (Table 1). Most patients in all groups were non-Hispanic, but a higher proportion of patients in the severely frail group were Hispanic compared to the non-frail group (5.1% vs. 3.8%, *p* < 0.001). BMI varied significantly among groups, with the proportion of obese patients increasing with increasing frailty, and the proportion of underweight patients decreasing with increasing frailty (*p* < 0.001). There were no significant differences between frailty groups for receipt of pre-operative mechanical bowel prep, receipt of pre-operative oral antibiotic prep, receipt of chemotherapy within 90 days, AJCC stage, operative approach, or total operation time.

### 3.2. Post-Operative Outcomes by Frailty

**Figure 1:**
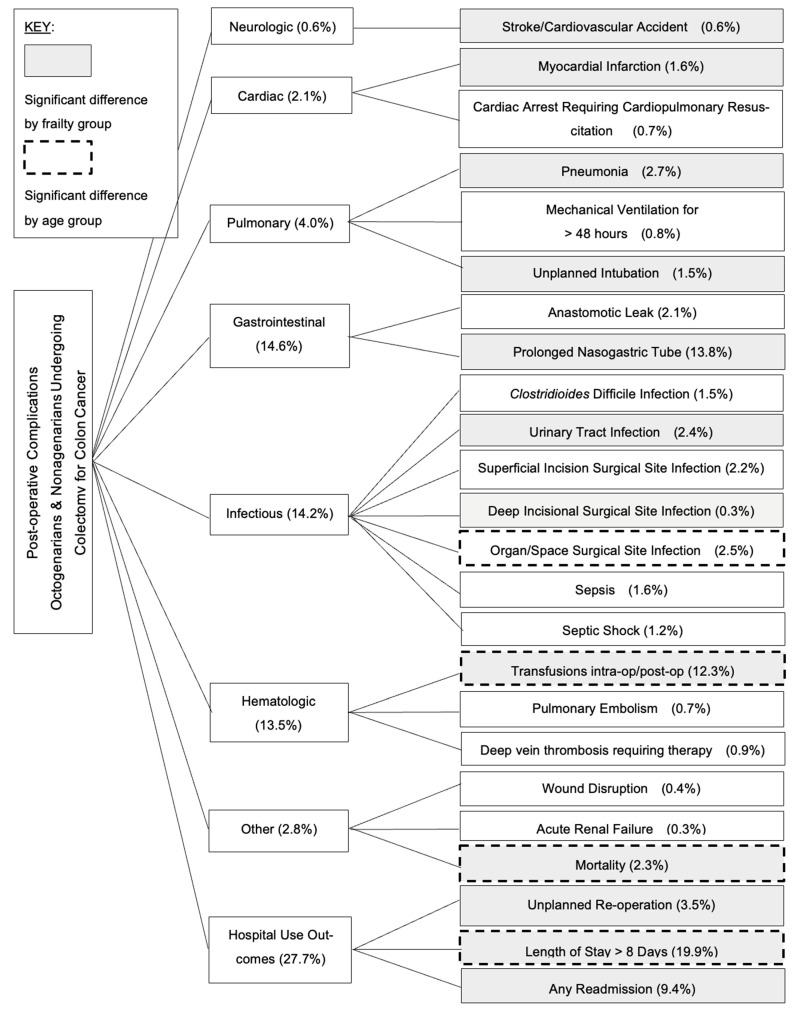
Post-operative outcomes and complications of octogenarians and nonagenarians undergoing elective colectomy for colon cancer.

Post-operative outcomes categorized by frailty group are described in Table 2. Length of hospital stay > 8 days was seen in greater proportions in mildly frail (19.1%) and severely frail (24.1%) patients, compared to non-frail patients (16.5%) (*p* < 0.001). The mortality rate was significantly higher in severely frail (3.4%) patients compared to non-frail (1.6%) and mildly frail (2.0%) patients (*p* < 0.001). Severely frail patients also had higher rates of readmission (10.8%) than non-frail (7.1%) and mildly frail (9.7%) patients (*p* < 0.001). Patients with increasing frailty had higher rates of neurologic complications (stroke or cerebrovascular accident), some pulmonary complications (pneumonia, unplanned intubation), and some cardiac complications (myocardial infarction). However, rates of mechanical ventilation for longer than 48 hours and cardiac arrest requiring CPR were not significantly different across frailty groups. Urinary tract infections were most frequent in the highest frailty group (*p* = 0.011). However, for most infectious complications the trend with frailty was not consistent or there were no significant differences between groups (superficial incisional SSI, deep incisional SSI, organ/space SSS, C. diff, sepsis, septic shock). Patients with increasing frailty had higher rates of prolonged nasogastric tube use (*p* < 0.001), need for intra-operative/post-operative blood transfusions (*p* < 0.001), and unplanned re-operation (*p* = 0.024). There were no significant differences in rates of anastomotic leak, wound disruption, acute renal failure, pulmonary embolism, or deep vein thrombosis. The outcomes and complications grouped by organ system are depicted in Figure 1.

### 3.3. Post-Operative Outcomes by Age

The post-operative outcomes categorized by age group are described in Table 3. When outcomes were stratified by age, nonagenarian patients had a higher rate of mortality than octogenarians (3.2% vs. 2.2%, *p* = 0.026). Nonagenarians were also more likely to have a length of stay > 8 days (23.1% vs. 19.4%, *p* = 0.002) but were not more likely than octogenarians to have unplanned operations (2.6% vs. 3.6%, *p* = 0.081) or readmissions (8.9% vs. 9.5%, *p* = 0.477). Nonagenarians were more likely to require intra-operative/post-operative transfusions than octogenarians (14.2% vs. 12.0%, *p* = 0.025). In contrast, nonagenarians were less likely than octogenarians to have organ/space SSIs (1.6% vs. 2.6%, *p* = 0.026). Neurologic, cardiac, pulmonary, gastrointestinal, other infectious, and other hematologic complications did not differ between octogenarians and nonagenarians.

### 3.4. Multivariate Analysis

After controlling for confounding variables, the nonagenarian age group was significantly associated with long length of stay > 8 days (aOR 1.36, 95% CI 1.13–1.63). However, unplanned re-operation, unplanned intubation, readmission, and mortality did not significantly differ between octogenarians and nonagenarians (Table 4 and Table 5). Severe frailty was significantly associated with unplanned intubation (aOR 2.41, 95% CI 1.27–4.59), long length of stay (aOR 1.73, 95% CI 1.44–2.09), readmission (aOR 1.84, 95% CI 1.42–2.39), and mortality (aOR 1.95, 95% CI 1.20–3.15). Severe frailty was not associated with unplanned re-operation. Mild frailty was associated with long length of stay (aOR 1.26 95% CI 1.06–1.50) and readmission (aOR 1.57, 95% CI 1.24–2.00), but not unplanned re-operation, unplanned intubation, or mortality. Male sex was associated with an increased risk of long length of stay and mortality compared to female patients. Having an underweight BMI was associated with an increased risk of mortality compared to normal weight BMI. Total OR time was associated with long length of stay and unplanned re-operation. Pre-operative oral antibiotics were significantly associated with a reduced risk of unplanned intubation and a reduced risk of long length of stay. Ethnicity, chemotherapy within 90 days, pre-operative mechanical bowel prep, and clinical stage were not associated with any of the tested outcomes.

## 4. Discussion

Our study found that among octogenarians and nonagenarians undergoing elective colectomy for colon cancer, frailty had a greater impact on post-operative morbidity and mortality than age alone. Increasing frailty was associated with higher mortality, increased readmission rates, and prolonged hospital stays. Severe frailty (mFI-5 of 2 or more) independently predicted mortality, long length of stay, unplanned intubation, and readmission, while being ≥ 90 years was independently associated with only long length of stay. Overall, frailty was significantly associated with 12 of the 24 complications in this study, while age was only significantly associated with 4 complications. This indicates that frailty may be a better predictor than age of morbidity and mortality after colectomy in the octogenarian and nonagenarian population. This study contributes to the existing literature by focusing specifically on octogenarians and nonagenarians undergoing colectomy for colon cancer and the impact of frailty on post-operative outcomes.

Prior work has examined post-operative colectomy outcomes in frail patients. However, few studies have focused on the very elderly. Sibia et al. used NSQIP to compare post-operative colectomy outcomes of frail vs. non-frail patients of all ages, and found that frail patients had an increased risk of superficial SSI, pneumonia, unplanned intubation, ventilator requirements for longer than 48 h, acute renal failure, UTI, stroke, MI, transfusion, DVT, re-operation and readmission than non-frail patients but did not find a difference in rates of sepsis [14]. Notably, in octogenarians and nonagenarians in our study, there were no differences in rates of superficial SSI, ventilator requirements for longer than 48 hours, acute renal failure, or DVT, differing from the above findings. The mean age in the study by Sibia et al. was 64.3, and the mean age of frail patients was 69.6, which may have influenced the differences in outcomes between the two studies. This highlights the idea that frail very elderly patients behave differently than their younger, frail counterparts, and supports the utility of our study. Both our study and Sibia et al. found that severe frailty (mFI-5 of 2 or more) was associated with readmission but not associated with re-operation in multivariate analysis. Interestingly, a study by Spence et al. found that age and American Society of Anesthesiologists (ASA) classification were better predictors of anastomotic leak than frailty, which is concordant with our finding that anastomotic leak was not associated with frailty [15]. However, it should be noted that the 2.1% rate of anastomotic leak found in our study was lower than the anastomotic leak rate reported in a systematic review and meta-analysis of 3.4% [16]. In our study, nonagenarians had an even lower anastomotic leak rate of 1.6%. This very low rate is consistent with a Dutch study which demonstrated that anastomotic leak rates after colectomy for colon cancer decreased as age increased, with the lowest rate in patients over 80 years old [17]. The same study found that mortality after anastomotic leak did increase with increasing age. Patient selection and surgical decision making such as avoiding primary anastomoses and opting to perform end colostomies in high-risk elderly patients are potential explanations for this lower rate in very elderly patients, but further research is required to validate these explanations.

Most studies of frailty in patients undergoing colectomy have utilized the mFI-5 [12,13,14]. However, several studies have used other frailty measures. For example, Obeid et al., whose study utilized an 11-factor modified frailty index based on the Canadian Study of Health and Aging Frailty Index, found that frail patients had higher rates of Clavien–Dindo class IV and V complications [18]. Another study by Normann et al., which utilized the Clinical Frailty Scale, found that frail patients aged 70 and older had higher 90-day and 1-year mortality rates [19]. Several studies have compared frailty scores in the elderly population. One recent study by Fagard et al. prospectively compared seven frailty screening instruments in 172 patients aged 70 and older and found that the Geriatric-8 (G8) score showed higher sensitivity and negative predictive value than the mFI-5 and other scores; however, the mFI-5 and other scores were more specific. The authors concluded that no single tool was clearly superior and recommended that comprehensive geriatric assessment, rather than frailty screening alone, should be used to assess post-operative risk [20]. Another study of 269 patients aged 70 and older with cancer who underwent elective abdominal surgery found that the G8 was the best predictor of 30-day morbidity and mortality [21]. The American Society of Colon and Rectal Surgeons (ASCRS) recommends considering frailty rather than chronological age in surgical decision making and endorses the use of the G8, mFI-5, and mFI-11 [22]. However, comparison of multiple frailty scoring systems specifically in the octogenarian and nonagenarian population undergoing colectomy for colon cancer has not been conducted and is an area for further research.

Both mild and severe frailty were significantly associated with long length of stay (hospital stay longer than 8 days) in our study. These prolonged hospital stays in frail patients not only reflect higher rates of complications but also carry significant implications for healthcare utilization and cost. In a large national cohort study of adults undergoing elective colectomy, frailty was associated with a USD 13,400 increment in hospitalization costs [23]. This underscores the importance of early identification of frail patients, careful decision making around surgical management, and targeted interventions to reduce complications to not only improve patient recovery but also reduce the financial burden on patients, families, and the healthcare system. One area that our study did not closely investigate was the influence of operative approach on post-operative outcomes in very elderly patients undergoing colectomy. In NSQIP, information about the surgical approach was missing for nearly 40% of the patients and thus we did not include it in our multivariate analyses. A study by Chok et al., which studied post-operative colectomy outcomes in patients 80 years of age and over, found that laparoscopic resection was associated with a decreased length of hospital stay compared to an open approach [24]. Another study by Lu et al. found that elderly patients undergoing right hemicolectomy for colon cancer had shorter lengths of hospital stay when a robotic-assisted approach rather than a laparoscopic approach was used [25]. This study focused on octogenarians and nonagenarians, but did not consider frailty, and there remains a need for further research into the influence of the operative approach on outcomes in frail, very elderly patients.

This study has several limitations. As a retrospective cohort study based on a large, administrative dataset, there is a predetermined number of variables that can be analyzed, and certain variables, such as presence of arrhythmia, post-operative ileus, delirium, and others are inevitably omitted. In addition, the NSQIP dataset tracks only 30-day outcomes after surgery, limiting the conclusions that can be drawn in terms of time frame. This invites future studies to investigate the effects of frailty on post-operative colectomy outcomes in frail elderly patients in the even longer term. Additionally, we excluded patients with hospital lengths of stay longer than 30 days, likely excluding patients with the most severe complications who may be the oldest or most frail. It is possible that this exclusion may have biased the results toward slightly healthier individuals and led to underestimation of the true impact of age and frailty on post-operative outcomes. Other inherent limitations of utilizing NSQIP include a potential loss to follow-up, with patients presenting to hospitals other than the index hospital or missed comorbidities that were not captured by medical coding. Additionally, only including data from NSQIP hospitals may lead to selection bias for patients treated at hospitals with more robust quality improvement programs, potentially skewing toward better outcomes. Finally, the limitations of using mFI-5 as frailty as opposed to a more comprehensive frailty measure like the Clinical Frailty Score include limited discrimination of more frail patients and omission of certain domains of frailty—such as cognitive ability and daily functioning. While these limitations exist, the latest ASCRS recommendations endorse the mFI-5 as a valuable marker of frailty [22].

## 5. Conclusions

This study found that frailty was independently associated with an increased risk of unplanned intubation, long length of stay, readmission, and mortality following colectomy in octogenarian and nonagenarians. These findings underscore the value of using frailty as a screening tool to guide surgical decision making and patient candidacy for surgery in the very elderly population.

## Figures and Tables

**Table 1:** Patient factors by frailty of patients undergoing elective colectomy for colon cancer in ACS-NSQIP database, 2015–2021.

	Overall N = 10,671	Non-frail (mFI-5 = 0) *N* = 2345 (22.0%)	Mildly Frail (mFI-5 = 1) *N* = 5482 (51.4%)	Severely Frail (mFI-5 = 2 or More) *N* = 2844 (26.7%)	*p*-Value
Age Group					<0.001 *
80–89 (octogenarian)	9412/10,671 (88.2)	2067/2345 (88.1)	4777/5482 (87.1)	2568/2844 (90.3)	
90+ (nonagenarian)	1259/10,671 (11.8)	278/2345 (11.9)	705/5482 (12.9)	276/2844 (9.7)	
Sex					<0.001 *
Female	5957/10,671 (55.8)	1331/2345 (56.8)	3140/5482 (57.3)	1486/2844 (52.3)	
Male	4714/10,671 (44.2)	1014/2345 (43.2)	2342/5482 (42.7)	1358/2844 (47.7)	
Race					<0.001 *
AAPI	456/8270 (5.5)	88/1731 (5.1)	214/4305 (5.0)	154/2234 (6.9)	
Black	535/8270 (6.5)	67/1731 (3.9)	260/4305 (6.0)	208/2234 (9.3)	
White	7235/8270 (87.5)	1564/1731 (90.4)	3815/4305 (88.6)	1856/2234 (83.1)	
Other	44/8270 (0.5)	12/1731 (0.7)	16/4305 (0.4)	16/2234 (0.7)	
Hispanic Ethnicity					<0.001 *
Yes	309/8383 (3.7)	67/1754 (3.8)	126/4340 (2.9)	116/2289 (5.1)	
No	8074/8383 (96.3)	1687/1754 (96.2)	4214/4340 (97.1)	2173/2289 (94.9)	
BMI					<0.001 *
Normal Weight 18.5–24.9	4045/10,671 (37.9)	1126/2345 (48.0)	2073/5482 (37.8)	846/2844 (29.7)	
Underweight <18.5	384/10,671 (3.6)	144/2345 (6.1)	162/5482 (3.0)	78/2844 (2.7)	
Overweight 25.0–29.9	3892/10,671 (36.5)	757/2345 (32.3)	2092/5482 (38.2)	1047/2844 (36.8)	
Obese >29.9	2346/10,671 (22.0)	318/2345 (13.6)	1155/5482 (21.1)	873/2844 (30.7)	
Pre-operative Mechanical Bowel Prep					0.611
Yes	6836/9462 (72.2)	1498/2098 (71.4)	3504/4830 (72.5)	1834/2534 (72.4)	
No	2626/9462 (27.8)	600/2098 (28.6)	1326/4830 (27.5)	700/2534 (27.6)	
Pre-operative Oral Antibiotic Prep					0.368
Yes	5687/9584 (59.3)	1228/2117 (58.0)	2935/4914 (59.7)	1524/2553 (59.7)	
No	3897/9584 (40.7)	889/2117 (42.0)	1979/4914 (40.3)	1029/2553 (40.3)	
Chemotherapy within 90 Days					0.106
Yes	402/10,560 (3.8)	103/2316 (4.4)	206/5435 (3.8)	93/2809 (3.3)	
No	10,158/10,560 (96.2)	2213/2316 (95.6)	5229/5435 (96.2)	2716/2809 (96.7)	
Clinical Stage					0.152
Stage 0	176/10,148 (1.7)	39/2237 (1.7)	90/5197 (1.7)	47/2714 (1.7)	
Stage I	2278/10,148 (22.4)	464/2237 (20.7)	1196/5197 (23.0)	618/2714 (22.8)	
Stage II	4399/10,148 (43.3)	984/2237 (44.0)	2279/5197 (43.9)	1136/2714 (41.9)	
Stage III	3295/10,148 (32.5)	750/2237 (33.5)	1632/5197 (31.4)	913/2714 (33.6)	
Operative Approach					0.548
Laparoscopic	4356/6483 (67.2)	949/1402 (67.7)	2297/3393 (67.7)	1110/1688 (65.8)	
Robotic	727/6483 (11.2)	147/1402 (10.5)	376/3393 (11.1)	204/1688 (12.1)	
Open	1400/6483 (21.6)	306/1402 (21.8)	720/3393 (21.2)	374/1688 (22.2)	
Total Operation Time					
Median [IQR]	150 [110, 204]	150 [112, 203]	149 [108, 202]	152 [112, 207]	0.104

* *p* < 0.05; Abbreviations: ACS-NSQIP = American College of Surgeons National Surgery Quality Improvement Program, mFI-5 = modified 5-factor frailty index, AAPI = Asian American or Pacific Islander, BMI = body mass index, IQR = interquartile range.

**Table 2:** Post-operative outcomes by frailty of patients undergoing elective colectomy for colon cancer in ACS-NSQIP database, 2015–2021.

	Overall N = 10,671	Non-frail (mFI-5 = 0) N = 2345 (22.0%)	Mildly Frail (mFI-5 = 1) N = 5482 (51.4%)	Severely Frail (mFI-5 = 2 or More) N = 2844 (26.7%)	*p*-Value
Anastomotic Leak	219/10,652 (2.1)	44/2343 (1.9)	108/5471 (2.0)	67/2838 (2.4)	0.394
Prolonged Nasogastric Tube	1465/10,645 (13.8)	257/2339 (11.0)	773/5471 (14.1)	435/29,835 (15.3)	<0.001 *
Superficial Incisional SSI	236/10,671 (2.2)	58/2345 (2.5)	106/5482 (1.9)	72/2844 (2.5)	0.132
Deep Incisional SSI	29/10,671 (0.3)	9/2345 (0.4)	8/5482 (0.1)	12/2844 (0.4)	0.036 *
Organ/Space SSI	268/10,671 (2.5)	56/2345 (2.4)	125/5482 (2.3)	87/2844 (3.1)	0.090
Wound Disruption	42/10,671 (0.4)	9/2345 (0.4)	23/5482 (0.4)	10/2844 (0.4)	0.892
Pneumonia	286/10,671 (2.7)	48/2345 (2.0)	143/5482 (2.6)	95/2844 (3.3)	0.015 *
Unplanned Intubation	159/10,671 (1.5)	24/2345 (1.0)	78/5482 (1.4)	57/2844 (2.0)	0.012 *
Pulmonary Embolism	79/10,671 (0.7)	14/2345 (0.6)	41/5482 (0.7)	24/2844 (0.8)	0.584
Mechanical Ventilation for > 48 h	82/10,671 (0.8)	14/2345 (0.6)	39/5482 (0.7)	29/2844 (1.0)	0.174
Acute Renal Failure	37/10,671 (0.3)	7/2345 (0.3)	18/5482 (0.3)	12/2844 (0.4)	0.713
Urinary Tract Infection	260/10,671 (2.4)	55/2345 (2.3)	115/5482 (2.1)	90/2844 (3.2)	0.011 *
Stroke/CVA	62/10,671 (0.6)	6/2345 (0.3)	23/5482 (0.4)	33/2844 (1.2)	<0.001 *
Cardiac Arrest Requiring CPR	71/10,671 (0.7)	8/2345 (0.3)	38/5482 (0.7)	25/2844 (0.0)	0.056
Myocardial Infarction	167/10,671 (1.6)	22/2345 (0.9)	93/5482 (1.7)	53/2844 (1.9)	0.011 *
Transfusions Intra-op/Post-op	1310/10,671 (12.3)	232/2345 (9.9)	663/5482 (12.1)	415/2844 (14.6)	<0.001 *
DVT Requiring Therapy	91/10,671 (0.9)	14/2345 (0.6)	47/5482 (0.9)	30/2844 (1.1)	0.203
Sepsis	173/10,671 (1.6)	30/2345 (1.3)	87/5482 (1.6)	56/2844 (2.0)	0.141
Septic Shock	125/10,671 (1.2)	22/2345 (0.9)	59/5482 (1.1)	44/2844 (1.5)	0.082
C. diff	156/10,671 (1.5)	27/2345 (1.2)	72/5482 (1.3)	57/2844 (2.0)	0.061
Unplanned Re-operation	370/10,671 (3.5)	60/2345 (2.6)	206/5482 (3.8)	104/2844 (3.7)	0.024 *
Any Readmission	1008/10,671 (9.4)	166/2345 (7.1)	534/5482 (9.7)	308/2844 (10.8)	<0.001 *
Length of Hospital Stay > 8 Days	2119/10,671 (19.9)	388/2345 (16.5)	1046/5482 (19.1)	685/2844 (24.1)	<0.001 *
Mortality	245/10,671 (2.3)	38/2345 (1.6)	110/5482 (2.0)	97/2844 (3.4)	<0.001 *

* *p* < 0.05; Abbreviations: ACS-NSQIP = American College of Surgeons National Surgery Quality Improvement Program, mFI-5 = modified 5-factor frailty index, SSI = surgical site infection, CVA = cerebral vascular accident, CPR = cardiopulmonary resuscitation, DVT = deep vein thrombosis, C. diff = *Clostridioides* difficile.

**Table 3:** Post-operative outcomes by age of patients undergoing elective colectomy for colon cancer in ACS-NSQIP database, 2015–2021.

	Overall N = 10,671	Age 80–89 N = 9412 (88.2%)	Age 90+ and Older N = 1259 (11.8%)	*p*-Value
Anastomotic Leak	219/10,671 (2.1)	199/9412 (2.1)	20/1259 (1.6)	0.219
Prolonged Nasogastric Tube	1465/10,645 (13.8)	1298/9412 (13.8)	167/1259 (13.3)	0.610
Superficial Incisional SSI	236/10,671 (2.2)	206/9412 (2.2)	30/1259 (2.4)	0.660
Deep Incisional SSI	29/10,671 (0.3)	25/9412 (0.3)	4/1259 (0.3)	0.739
Organ/Space SSI	268/10,671 (2.5)	248/9412 (2.6)	20/1259 (1.6)	0.026 *
Wound Disruption	42/10,671 (0.4)	38/9412 (0.4)	4/1259 (0.3)	0.647
Pneumonia	286/10,671 (2.7)	243/9412 (2.6)	43/1259 (3.4)	0.085
Unplanned Intubation	159/10,671 (1.5)	144/9412 (1.5)	15/1259 (1.2)	0.352
Pulmonary Embolism	79/10,671 (0.7)	73/9412 (0.8)	6/1259 (0.5)	0.245
Mechanical Ventilation for > 48 h	82/10,671 (0.8)	77/9412 (0.8)	5/1259 (0.4)	0.108
Acute Renal Failure	37/10,671 (0.3)	36/9412 (0.4)	1/1259 (0.1)	0.086
Urinary Tract Infection	260/10,671 (2.4)	227/9412 (2.4)	33/1259 (2.6)	0.651
Stroke/CVA	62/10,671 (0.6)	52/9412 (0.6)	10/1259 (0.8)	0.289
Cardiac Arrest Requiring CPR	71/10,671 (0.7)	65/9412 (0.7)	6/1259 (0.5)	0.380
Myocardial Infarction	167/10,671 (1.6)	145/9412 (1.5)	22/1259 (1.7)	0.579
Transfusions Intra-op/Post-op	1310/10,671 (12.3)	1131/9412 (12.0)	179/1259 (14.2)	0.025 *
DVT Requiring Therapy	91/10,671 (0.9)	77/9412 (0.8)	14/1259 (1.1)	0.287
Sepsis	173/10,671 (1.6)	155/9412 (1.6)	18/1259 (1.4)	0.567
Septic Shock	125/10,671 (1.2)	105/9412 (1.1)	20/1259 (1.6)	0.143
C. diff	156/10,671 (1.5)	134/9412 (1.4)	22/1259 (1.7)	0.665
Unplanned Re-operation	370/10,671 (3.5)	337/9412 (3.6)	33/1259 (2.6)	0.081
Any Readmission	1008/10,671 (9.4)	896/9412 (9.5)	112/1259 (8.9)	0.477
Length of Hospital Stay > 8 Days	2119/10,671 (19.9)	1828/9412 (19.4)	291/1259 (23.1)	0.002 *
Mortality	245/10,671 (2.3)	205/9412 (2.2)	40/1259 (3.2)	0.026 *

* *p* < 0.05; Abbreviations: ACS-NSQIP = American College of Surgeons National Surgery Quality Improvement Program, SSI = surgical site infection, CVA = cerebrovascular accident, CPR = cardiopulmonary resuscitation, DVT = deep vein thrombosis, C. diff = *Clostridioides* difficile.

**Table 4:** Regression models for unplanned re-operation, unplanned intubation, and mortality.

	Unplanned Re-Operation		Unplanned Intubation		Mortality	
	aOR (95% CI)	*p*-Value	aOR (95% CI)	*p*-Value	aOR (95% CI)	*p*-Value
Age (years)						
80–89 (octogenarian)	Ref	-	Ref	-	Ref	-
90+ (nonagenarian)	0.88 (0.58–1.36)	0.575	0.88 (0.47–1.67)	0.700	1.75 (1.13–2.70)	0.012
Sex						
Female	Ref	-	Ref	-	Ref	-
Male	1.39 (1.06–1.83)	0.018	1.49 (1.00–2.23)	0.049	1.87 (1.33–2.64)	<0.001 *
Race						
Black	1.08 (0.48–2.41)	0.859	1.25 (0.43–3.64)	0.684	2.39 (0.86–6.61)	0.093
Other	1.04 (0.13–8.30)	0.972	0.82 (0.48–1.42)	0.482	6.64 (1.25–32.39)	0.027
AAPI	Ref	-	Ref	-	Ref	-
White	1.38 (0.77–2.47)	0.285	0.85 (0.54–1.35)	0.494	2.18 (0.94–5.07)	0.071
Ethnicity						
Hispanic	1.13 (0.52–2.45)	0.759	1.91 (0.76–4.82)	0.168	0.66 (0.21–2.12)	0.487
Non-Hispanic	Ref	-	Ref	-	Ref	-
BMI						
Normal weight 18.5–24.9	Ref	-	Ref	-	Ref	-
Underweight < 18.5	0.85 (0.39–1.86)	0.682	1.18 (0.42–3.36)	0.756	3.76 (2.06–6.88)	<0.001 *
Overweight 25.0–29.9	0.84 (0.62–1.13)	0.246	0.85 (0.54–1.35)	0.494	1.00 (0.67–1.47)	0.983
Obese > 29.9	0.52 (0.35–0.79)	0.002 *	0.82 (0.48–1.42)	0.482	0.79 (0.48–1.29)	0.418
Chemotherapy within 90 days						
Yes	0.70 (0.32–1.53)	0.374	0.44 (0.11–1.81)	0.253	1.28 (0.58–2.82)	0.542
No	Ref	-	Ref	-	Ref	-
Pre-operative mechanical bowel prep						
Yes	0.78 (0.55–1.10)	0.160	1.37 (0.82–2.30)	0.229	0.84 (0.56–1.28)	0.418
No	Ref	-	Ref	-	Ref	-
Pre-operative oral antibiotic prep						
Yes	0.92 (0.67–1.28)	0.631	0.47 (0.30–0.73)	<0.001 *	0.61 (0.42–0.90)	0.013
No	Ref	-	Ref	-	Ref	-
Clinical stage						
Stage 0	Ref	-	Ref	-	Ref	-
Stage I	1.92 (0.46–8.10)	0.374	0.70 (0.16–3.09)	0.639	1.27 (0.29–5.50)	0.749
Stage II	2.24 (0.54–9.30)	0.266	0.88 (0.21–3.75)	0.866	1.24 (0.29–5.27)	0.766
Stage III	2.08 (0.50–8.66)	0.317	0.76 (0.18–3.29)	0.718	1.60 (0.38–6.78)	0.525
mFI-5						
0	Ref	-	Ref	-	Ref	-
1	1.49 (1.02–2.18)	0.040	1.68 (0.90–3.10)	0.101	1.10 (0.69–1.75)	0.702
2 or more	1.57 (1.03–2.39)	0.036	2.41 (1.27–4.59)	0.007 *	1.95 (1.20–3.15)	0.007 *
Total OR time	1.00 (1.00–1.00)	0.001 *	1.00 (1.00–1.01)	0.053	1.00 1.00–1.00)	0.626

* *p* < 0.01; Abbreviations: aOR = adjusted odds ratio, CI = confidence interval; AAPI = Asian American or Pacific Islander; BMI = body mass index; mFI-5 = modified 5-factor frailty index; OR = operating room.

**Table 5:** Regression models for long length of stay (> 8 days) and readmission.

	Long Length of Stay (>8 days)		Readmission	
	aOR (95% CI)	*p*-Value	aOR (95% CI)	*p*-Value
Age (years)				
80–89	Ref	-	Ref	-
90+	1.36 (1.13–1.63)	0.001 *	0.94 (0.73–1.22)	0.639
Sex				
Female	Ref	-	Ref	-
Male	1.28 (1.12–1.45)	<0.001 *	1.08 (0.92–1.28)	0.344
Race				
Black	0.88 (0.64–1.22)	0.446	1.49 (0.96–2.32)	0.076
Other	0.18 (0.04–0.75)	0.019	1.11 (0.37–3.33)	0.849
AAPI	Ref	-	Ref	-
White	0.71 (0.56–0.90)	0.005 *	1.10 (0.77–1.57)	0.595
Ethnicity				
Hispanic	1.02 (0.69–1.49)	0.939	1.32 (0.83–2.08)	0.243
Non-Hispanic	Ref	-	Ref	-
BMI				
Normal weight 18.5–24.9	Ref	-	Ref	-
Underweight <18.5	1.49 (1.08–2.05)	0.015	1.32 (0.86–2.03)	0.199
Overweight 25.0–29.9	0.94 (0.81–1.09)	0.438	1.07 (0.88–1.29)	0.500
Obese >29.9	0.97 (0.81–1.15)	0.719	0.94 (0.75–1.19)	0.620
Chemotherapy within 90 days				
Yes	1.02 (0.74–1.40)	0.921	1.11 (0.75–1.66)	0.603
No	Ref	-	Ref	-
Pre-operative mechanical bowel prep				
Yes	0.91 (0.78–1.08)	0.281	1.05 (0.84–1.32)	0.645
No	Ref	-	Ref	-
Pre-operative oral antibiotic prep				
Yes	0.61 (0.52–0.70)	<0.001 *	0.84 (0.69–1.02)	0.082
No	Ref	-	Ref	-
Clinical stage				
Stage 0	Ref	-	Ref	-
Stage I	1.08 (0.63–1.84)	0.782	0.66 (0.37–1.18)	0.161
Stage II	1.33 (0.79–2.25)	0.286	0.88 (0.50–1.54)	0.648
Stage III	1.56 (0.92–2.64)	0.098	0.74 (0.42–1.31)	0.300
mFI-5				
0	Ref	-	Ref	-
1	1.26 (1.06–1.50)	0.008 *	1.57 (1.24–2.00)	<0.001 *
2 or more	1.73 (1.44–2.09)	<0.001 *	1.84 (1.42–2.39)	<0.001 *
Total OR time	1.00 (1.00–1.00)	<0.001 *	1.001(1.00–1.00)	0.019

* *p* < 0.01; Abbreviations: aOR = adjusted odds ratio, CI = confidence interval; AAPI = Asian American or Pacific Islander; BMI = body mass index; mFI -5= modified 5-factor frailty index; OR = operating room.

## Data Availability

The data used in this study were obtained from the American College of Surgeons National Surgical Quality Improvement Program (ACS NSQIP) colectomy-targeted dataset for the years 2015 to 2021. These data are not publicly available due to privacy and ethical restrictions, but access can be requested through the American College of Surgeons NSQIP Participant Use File (PUF) program for eligible institutions.

## Abbreviations

The following abbreviations are used in this manuscript:

NSQIP	National Surgery Quality Improvement Program
mFI-5	Five-factor modified frailty index
ACS	American College of Surgeons
BMI	Body mass index
AJCC	American Joint Committee on Cancer
DM	Diabetes mellitus
CHF	Congestive heart failure
COPD	Chronic obstructive pulmonary disease
AAPI	Asian American and Pacific Islander
SSI	Surgical site infection
C. diff	*Clostridioides* difficile
PE	Pulmonary embolism
UTI	Urinary tract infection
CVA	Cerebrovascular accident
CPR	Cardiopulmonary resuscitation
MI	Myocardial infraction
DVT	Deep vein thrombosis
aOR	Adjusted odds ratio
CI	Confidence interval
IQR	Interquartile range
OR	Operating room
ASA	American Society of Anesthesiologists
ICU	Intensive care unit
